# DETexT: An SNV detection enhancement for low read depth by integrating mutational signatures into TextCNN

**DOI:** 10.3389/fgene.2022.943972

**Published:** 2022-09-28

**Authors:** Tian Zheng

**Affiliations:** ^1^ Department of Computer Science and Technology, School of Electronic and Information Engineering, Xi’an Jiaotong University, Xi’an, China; ^2^ Institute of Data Science and Information Quality, Shaanxi Engineering Research Center of Medical and Health Big Data, Xi’an Jiaotong University, Xi’an, China

**Keywords:** extra-low read depth, variant detection, TextCNN, mutational signatures, SNV

## Abstract

Detecting SNV at very low read depths helps to reduce sequencing requirements, lowers sequencing costs, and aids in the early screening, diagnosis, and treatment of cancer. However, the accuracy of SNV detection is significantly reduced at read depths below ×34 due to the lack of a sufficient number of read pairs to help filter out false positives. Many recent studies have revealed the potential of mutational signature (MS) in detecting true SNV, understanding the mutational processes that lead to the development of human cancers, and analyzing the endogenous and exogenous causes. Here, we present DETexT, an SNV detection method better suited to low read depths, which classifies false positive variants by combining MS with deep learning algorithms to mine correlation information around bases in individual reads without relying on the support of duplicate read pairs. We have validated the effectiveness of DETexT on simulated and real datasets and conducted comparative experiments. The source code has been uploaded to https://github.com/TrinaZ/extra-lowRD for academic use only.

## 1 Introduction

Mutation detection is important for the accurate diagnosis and personalized therapeutics of cancer (Fanfani et al., 2021). Somatic mutations are caused by exogenous and endogenous mutational processes that operate during the cell lineage between the fertilized eggs and the cancer cells. Each mutational process may involve components of DNA damage or modification, DNA repair, and DNA replication (which may be normal or abnormal) and potentially include base substitutions, small insertions and deletions (indels), genome rearrangements, and chromosome copy number changes (Stratton et al., 2009). Of all mutation types, single nucleotide variants (SNVs) have high genetic stability, which helps to determine the relationship between genetic polymorphism and disease, explain the susceptibility to phenotypic differences between individuals, and have important implications for disease diagnosis, individualized treatment, and prognosis (Xu et al., 2012; Cashman et al., 2020).

Accurate detection of SNVs with low read depths is of great significance and helps in early cancer screening, diagnosis, and treatment (Kothen et al., 2018). Read depth is the ratio of the total number of bases to the size of the genome (Sims et al., 2014). Most SNV detection methods rely on high sequence read depth and false-positive results appear to increase with the decrease of sequencing depth (Xiao et al., 2021). Although advances in sequencing technologies have reduced the cost of high read depth, the effective depth remains low in the case of prenatal fetal detection (Alba et al., 2012), low purity tumors (Wilkerson et al., 2014), and subclonal structures (Al-Katib et al., 2020). For example, liquid biopsies that identify cancer mutations through blood material have been proposed as a transformative technology for early cancer screening and residual disease monitoring (Kleftogianniss et al., 2020). However, the proportion of ctDNA (cell-free tumor DNA) in the overall blood DNA is relatively low, particularly in situations of low disease burden, such as early cancer detection, and residual disease surveillance after therapeutic intervention ( Underhill., 2021).

The solution to this problem is not unambiguous. Existing methods, such as MuTect, have a sensitivity that drops below 0.1 at 5% variant allelic frequency (VAF) and ×10 read depth (Steven et al., 2018). The possible causes are analyzed in this study and listed as follows. 1) Existing methods mainly calculate reads mapped to the same allele as a set of extracted features to locate mutations (Fang et al., 2015). Their performance is limited in challenging situations such as low-complexity regions and low tumor purity (Steven et al., 2018; Esteva et al., 2019; Zheng et al., 2021). 2) Existing methods align each read pair to a haplotype to obtain a likelihood matrix based on the pairwise HMM algorithm and then use a Bayesian somatic likelihood model to obtain the log ratio of somatic mutation to sequencing error (Cibulskis et al., 2013). Mutations supported by low read depths are indistinguishable in the values of these models. The existing methods cannot support single reads and require many hard filters to filter mutation candidates (Sahraeian et al., 2019). Furthermore, existing deep learning-based methods treat the sequence reads as images for variant detection and encode bases by one-hot encoding (Poplin et al., 2018; Luo et al., 2019; Sahraeian et al., 2019). However, 1) this encoding method only extracts the information of the base itself in the convolution operation and does not notice the differences between the variant sites and other bases and 2) mutation identification using only two-dimensional information may take up less resource.

Recent studies have highlighted the potential of mutational signatures (MSs) for the accurate detection of SNVs (Lawrence et al., 2013; Alexandrov et al., 2020). Somatic mutations in the cancer genome are caused by multiple mutational processes, each producing features called MSs. Over the last few years, large-scale analyses have revealed many MSs in human cancer types. The Pan-Cancer Analysis of Whole Genomes (PCAWG) Network have analyzed data from over 23,000 samples (Tarabichi et al., 2021), showing that SNV tri-nucleotide structures have unique distribution characteristics. The observed MSs suggest that the occurrence of SNV is not an equiprobable event and may help to reveal the true probability of positive SNV, which provides a promising potential approach for SNV detection at low read depths.

Inspired by these, this study presents DETexT, an SNV detection enhancement model that works at very low read depths by integrating MS and the deep semantic learning model Text Convolution Neural Network (TextCNN). DETexT uses a training scheme that not only detects true mutations with high sensitivity but also rejects candidate mutations caused by systematic sequencing artifacts. It integrates MS to maximize the extraction of features carried by individual reads and reflects the cumulative effects of exogenous and endogenous mutational processes acting on cancer cells. Specifically, DETexT can be divided into three parts: 1) Read coding. It proposes a differential encoding algorithm that translates the difference information between reads and the reference genome into an input matrix for a deep learning model instead of a one-hot encoder. 2) TextCNN learning and training. 3) Integration of MS as prior probabilities. We conducted several experiments on simulated data and a dataset of esophageal cancer (ESCC) SNVs. The results show that DETexT can filter out the false-positive variant candidates at very low read depths, which may provide support for liquid biopsy technology for cancer.

## 2 Materials and methods

### 2.1 Overview of DETexT

**FIGURE 1:**
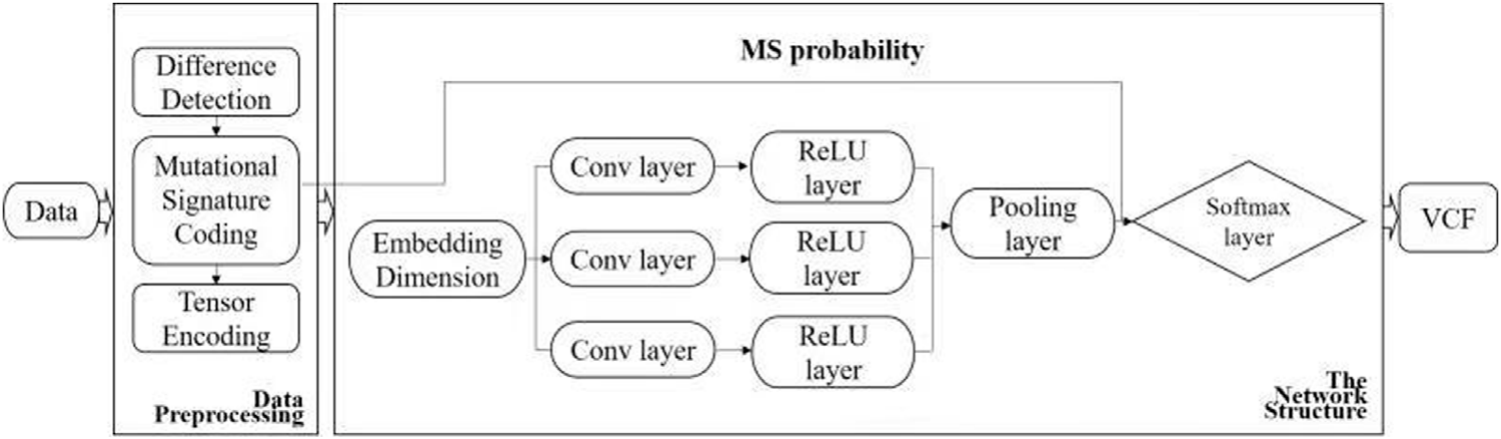
Workflow of DETexT. The workflow of DETexT can be divided into two parts: data pre-processing and network structure. 1) Data pre-processing includes different base detection, mutational signature encoding, and tensor encoding. 2) The network structure consists of three convolution filters, each followed by a ReLU layer. The mutational signature probabilities are applied before the Softmax layer. The output is the true SNV after the false positive filter.

**FIGURE 2:**
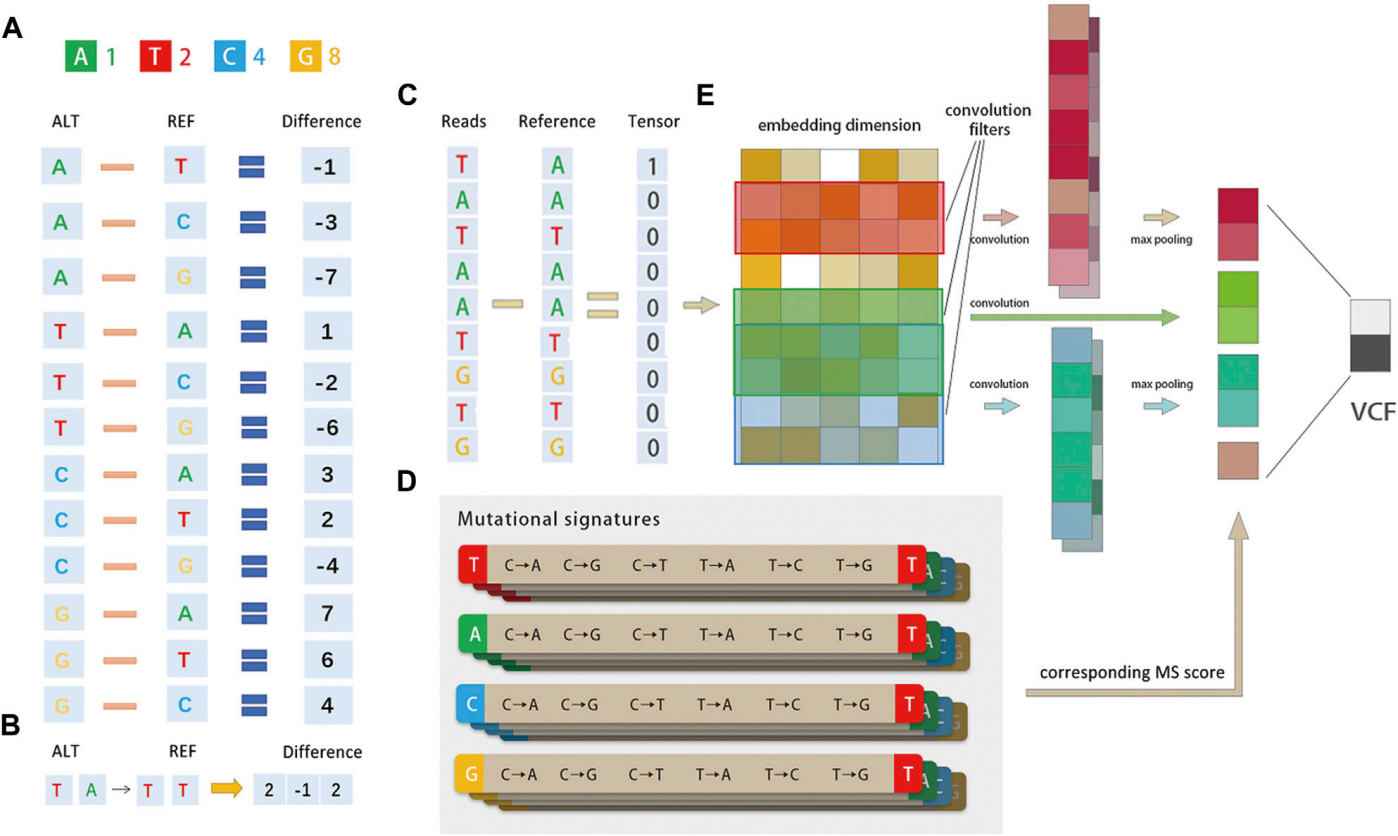
Method diagram. **(A)**Diagrammatic representation of the base difference. Bases A/T/C/G are converted to 1/2/4/8 codes and for each read, and the difference between it and the corresponding position of the built-in reference is calculated. The mutational signature is encoded by tri-nucleotide structure, for example, if a position is A → T, the corresponding position is encoded as 1–2 = −1. **(B)**Representation of the tri-nucleotide structure of the mutational signature. The mutational signature is encoded by tri-nucleotide structure; if a position has T (A→T) T, the corresponding position is encoded as (2, −1, 2). **(C)** Schematic representation of the entire pipeline. The network structure consists of three convolution filters, each followed by a ReLU layer. The mutational signature probabilities are concatenated before the Softmax layer. The output is the true SNV with false positives filtered. **(D)** There are 96 possible mutations as we incorporated six classes of base substitution: C > A, C > G, C > T, T > A, T > C, and T > G (referred by a pyramiding of mutated Watson–Crick base pair) and base information for the 5′ and 3′ bases immediately adjacent to each mutated base. **(E)** DETexT converts the reads of the variance representation **(C)** into the embedding_size dimension **(E)** through the embedding operation of the tensor.

The input to the proposed method is a sequence alignment map (SAM) file, the built-in reference is the human genome 19 (hg19), and the output is a variant calling format (VCF). The specific pipeline of the proposed method is shown in Figures 1, 2 and can be divided into three parts: 1) encoding of the read-pair difference representation, 2) learning and training of the TextCNN, and 3) integration of the MS as prior probabilities.

### 2.2 Difference detection and tensor encoding

DETexT devised the coding principle for the difference representation of sequencing data, as shown in Figure 2A. The read bases A/T/C/G are converted to 1/2/4/8, and the difference between each read base and the corresponding position of the built-in reference is calculated by numerical subtraction. For example, if a position has A → T, the corresponding position code is 1–2 = −1. One-hot CNNs may not be suitable for classification when one has a small to medium-sized training dataset, possibly due to sparsity: the reads of NGS are perhaps too brief to provide sufficient information for such high dimensional encoding (Zhang and Wallace., 2015). Compared to the one-hot coding algorithm used in existing methods, the replaced bases (−1, −2, −4, ±3, ±6...) may differ from the representation of ATCG bases (1/2/4/8), which retains the information before and after the base substitution. In addition, the information of one of the original bases around the variable point is also retained [i.e., encoding T (A→T) T as ( 2, −1, 2)], as shown in Figure 2B.

The user is supported to set the vector dimension manually according to the length of the input reads, with a default value of 100 (bps). DETexT converts the reads of difference representation (Figure 2C) into the embedding_size dimension (Figure 2E) by the embedding operation of the tensor. Here, the number of the embedding is 20 and the embedding size is 5.

### 2.3 Model selection and architecture

SNV detection on a single read is essentially a binary classification problem, that is, whether the difference between a read and the reference is a genuine mutation or an error that needs to be screened. Compare to traditional neural networks such as MLP, convolutional neural network has the characteristic of sparse interactions, parameter sharing, and equivariant representations. TextCNN (Sahraeian et al., 2019) is a successful application of image CNN networks on text data with a simple network structure, small number of parameters, low computational effort, and fast training. It can automatically combine and filter N-gram features to obtain semantic information at different levels of abstraction. These make it suitable for processing the sequence data. On a single-card v100 machine, it can train 1.65 million data, iterate 260,000 steps, and converge in about half an hour. Inspired by these, DETexT has proposed an attempt to detect SNV with the TextCNN model.

#### 2.3.1 Implementation details

The specific structure and parameters of DETexT are shown in Figure 2E; Table 1, and described below. The input of the TextCNN is the embedding layer computed in Section 2.2. We start with tokenized mutation candidate reads, which are then transformed into a vector representation of each token. The model architecture is a slight variant of the TextCNN architecture (Zhang and Wallace. 2015). The convolution layer here is one-dimensional. DETexT depicts three filter region sizes, namely, 2, 3, and 4, with one filter size of 100 and a step size of 1. Each filter has two output channels. DETexT uses rectified linear unit (ReLU) as the activation function and sets the pooling operation to 1-max pooling. The dropout rate is 0.5 and the l2 norm constraint is 3. The Softmax activation function is used to calculate the probability of each category (true variant or false positive).

**TABLE 1:** Baseline configuration.

Description	Values
Filter region size	(2, 3, 4)
Feature maps	100
Activation function	ReLU
Pooling	1-max pooling
Dropout rate	0.5
*l*2 norm constraint	3

#### 2.3.2 Feature extraction

**FIGURE 3:**
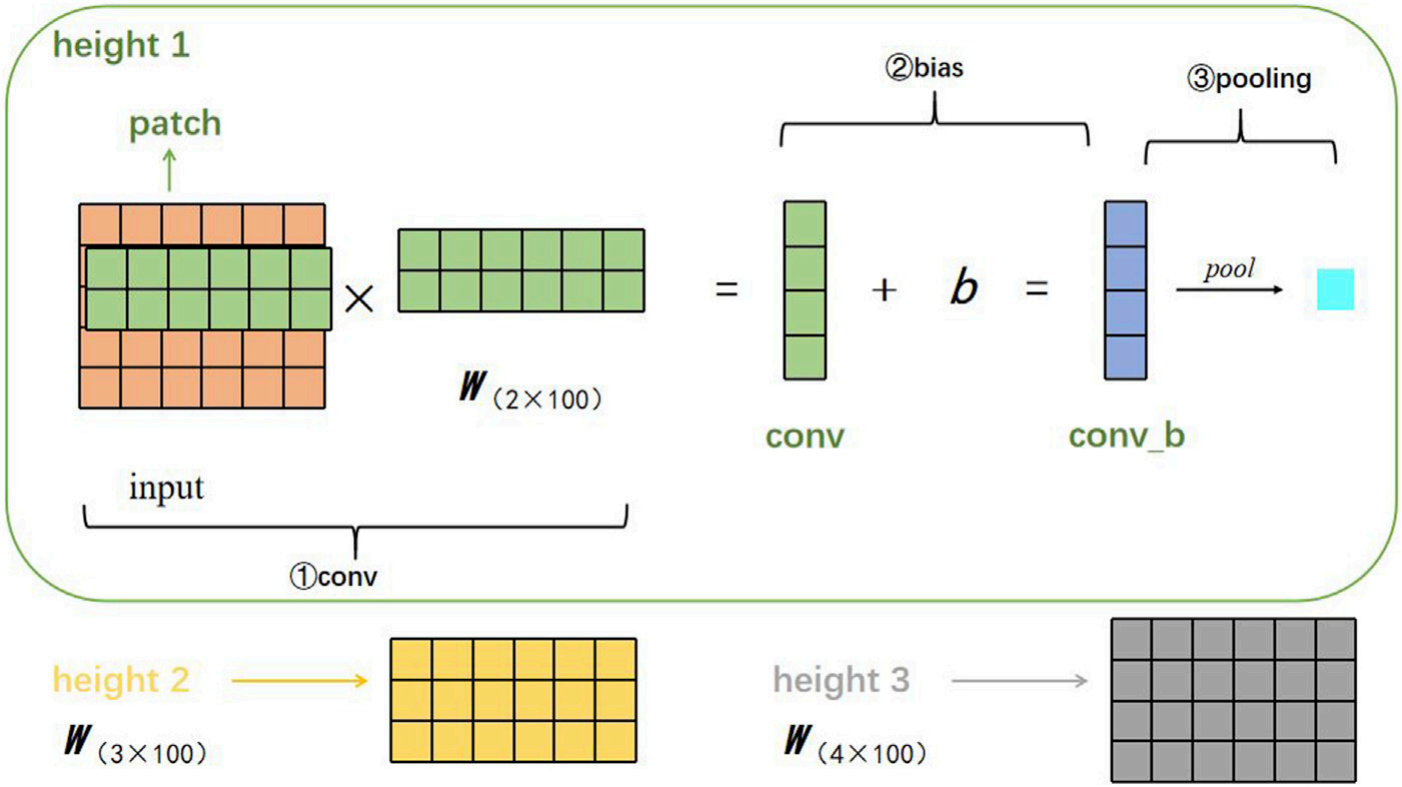
Visualization of the corresponding convolution calculation steps.

We describe here the process of feature extraction. Suppose there is a filter parameterized by a weight matrix w with region size *h*, then w will contain *h·d* parameters to be estimated. DETexT uses filters with widths equal to the read vectors dimension (*d* = 100) and simply varies the ‘height’ of the filter as the region size of the filter. Furthermore, since the embedding size is 5, we set the three region sizes (*h*) to (2, 3, 4), as shown in Figure 3.

We use $A\in {R\;}^{5\times 100}$ to denote the input matrix, and A[i : j] denotes the submatrix of A from the *i*th to *j*th rows. The output sequence $o\in {R\;}^{5-h+1}$ of the convolution operator is obtained by repeatedly applying the filter to the sub-matrices of A:

$$\begin{array}{c} o_{i}=w\cdot A\left[i:i+h-1\right],i=1,\;.\;.\;.,\;5-h+1, \end{array}$$ (1)

where ⋅ is the dot product (sum of multiplication of elements) between the submatrix and the filter. We add a bias term b∈R and an activation function *f* to each $o_{i}$ , including the feature map $c\in {R\;}^{5-h+1}$ for this filter:

$$c_{i}=f\left(o_{i}+b\right).$$ (2)

The feature map can be presented as follows:

$$c=\left[c_{1},\;c_{2},\;.\;.\;.\;,\;c_{5-h+1}\right].$$ (3)

We then apply a max-overtime pooling operation over the feature map and use the maximum value $\hat{c\;}=\max\left\{c\right\}$ as the feature corresponding to this particular filter. The idea is to capture the most important feature—one with the highest value—for each feature map. 1-max pooling is uniformly better than other pooling strategies (Zhang and Wallace. 2015).

#### 2.3.3 Regularization

These features form the penultimate layer and are passed to a fully connected softmax layer whose output is the probability distribution of the labels. For regularization, DETexT employs dropout in the penultimate layer to constrain the *l*_2_ norms of the weight vectors (Hinton et al., 2012). The dropout method prevents the hidden units from co-adaptation through random rejection, that is, a certain proportion of hidden units are set to zero during forward backpropagration. That is, given the penultimate layer $z=\left[{\hat{c}}_{1},\;\ldots ,\;{\hat{c}}_{3}\right]$ (note that here we have three filters), for the output unit *y* in the forward propagation, dropout uses

y=w⋅(z∘r)+b, (4)

where ∘ is the element-wise multiplication operator, and $r\in R^{3}$ is a ‘masked’ vector of Bernoulli random variables with a probability *p* of being 1. The gradients are backpropagated only through the unmasked units. At test time, the learned weight vectors are scaled by *p* such that $\hat{w}=pw$ , and $\hat{w}$ is used (without dropout) to score the unseen mutation candidate. We also constrain *l*_2_ norms of the weight vector by rescaling *w* so that after the gradient descent step, we have *||w||*_*2*_ *= s* whenever *||w||*_*2*_ *> s* (Yoon., 2014). We set the dropout rate (*p*) to 0.5 and the *l*_2_ norm constraint (*s*) to 3 based on the sensitivity analysis, as shown in Section 3.3.2.

### 2.4 Integration of mutational signatures

A third innovation of DETexT is the integration of mutational signatures (MSs), which are mutations types shared between patients or in local sequence environments that can reveal information about the somatic mutational process such as slight infidelity inherent in the DNA replication machinery, exposures to exogenous or endogenous mutagens, enzymatic modification, exposures to DNA repair defects or abnormalities maintenance (Lawrence et al., 2013; Alexandrov et al., 2020). MS have been tentatively identified by analyzing 4,938,362 somatic substitutions and small insertions/deletions (InDels) from the mutational catalogs of 7,042 primary cancers of 30 different classes (507 from whole genome and 6,535 from exome sequences) with the nonnegative matrix factorization (NMF) algorithm (Alexandrov et al., 2013).

There are 96 possible mutations as we incorporated six classes of base substitution: C > A, C > G, C > T, T > A, T > C, and T > G (referred to by the pyramiding of the mutated Watson–Crick base pair) as well as information on the 5′ and 3′ bases immediately adjacent to each mutated base, as shown in Figure 2D) (Alexandrov et al., 2013). The 96 substitution classifications are particularly useful for distinguishing MS that give rise to the same substitution but in different sequence contexts. Mutational processes from different etiologies are active during cancer development and can be identified by MS due to their unique mutational pattern and specific activity on the genome. The results reveal the diversity of mutational processes during the development of cancer and have potential implications for understanding cancer etiology (Tianyuan Liu et al., 2022), prevention (Aaron Chevalier et al., 2021), and therapy (Drews et al., 2022).

**FIGURE 4:**
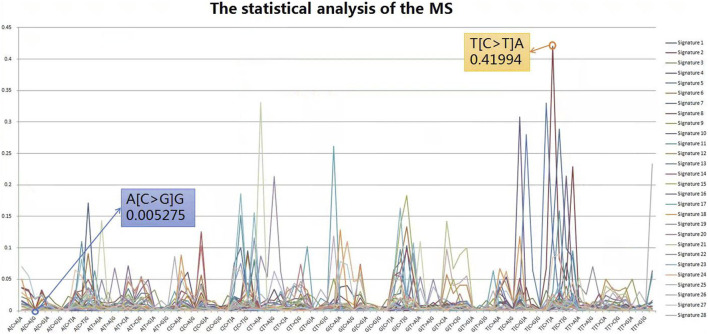
Statistical analysis of the MS. From the results of probability statistics, A (C > G) G has the lowest probability of occurrence among the 96 classifications at 0.005275 while T (C > T) A has the highest at 0.41994, a difference of 79.6 times.

The Catalogue Of Somatic Mutations In Cancer (COSMIC) database is the world’s largest and most comprehensive resource for exploring the impact of somatic mutations on human cancer. Extensive statistical analyses have shown that variable tri-nucleotide structures have distributional characteristics and that the probability of occurrence of sharp base substitution is not an equal probability event (Helleday et al., 2014; Roberts and Gordenin, 2014). The incidence of somatic mutations varies widely between and within cancer categories, from about 0.001 per megabase (Mb) to over 400 per Mb. Certain childhood cancers [Pilocytic astrocytoma (∼0.001), medulloblastoma (∼0.01), and kidney chromophobe (∼0.1)] have the fewest mutations, whereas cancers associated with chronic mutagenic exposures such as lung (tobacco smoking, ∼100) and malignant melanoma (exposure to ultraviolet light, ∼400) exhibited the highest prevalence (Alexandrov et al., 2013). This variation in mutation prevalence can be attributable to differences in cell lineage duration between the fertilized egg and the cancer cell being sequenced and/or differences in somatic mutation rates during all or part of that cell lineage (Stratton et al., 2009). From the results of the probability statistics, the probability of occurrence of A (C > G) G was 0.005275, the lowest of the 96 classifications, whereas T (C > T) A was the highest at 0.41994, a 79.6-fold difference, as shown in Figure 4. In conclusion, mutational signatures are summaries of mutation occurrence rules extracted from large-scale cancer data, and it is necessary to judge variants with mutational signatures suggesting high probability among candidate mutations as true mutations, rather than false positives.

DETexT downloaded the mutation signature probability from the COSMIC, traversed the probability value of the 96 classifications, and integrated the maximum occurrence probability of each mutational signature as prior knowledge into the deep learning framework to help filter mutation false positives, as shown in Figure 2C. DETexT matches the candidate mutation to 96 types and obtains the probability values from the COSMIC. We took the prior value of the maximum occurrence probability, added it to the convolution kernel processing result, extended the convolution kernel result to one dimension, and put them into a Softmax layer for training to help obtain the classification probability values. Section 3 presents the experimental results demonstrating that the design can greatly improve the accuracy of mutation detection. When there is no agglomeration effect of read pairs at very low read depth, this method can maximize the retention of essential information about candidate mutation and filter out the false positives. Furthermore, during the integration of mutational signatures, we tested the difference between adding the probability directly and multiplying by a coefficient, considering the numerical difference between the probability value and convolution kernel results, and determined the coefficient to seven based on experimental verification, as shown in Section 3.3.1.

## 3 Experiments and results

We conducted a series of experiments on several simulated and real datasets covering esophageal cancer (ESCC) and benchmark datasets to validate the performance of DETexT. How we obtained the data and performance is described in detail in each section. In Section 3.1, we describe the datasets and conduct experiments on test and training datasets of different sizes. In Section 3.2, we test the performance of DETexT on 10 simulated datasets and compare it with existing advanced detection software and algorithms. In Section 3.3, we test the performance of the DETexT on ESCC real datasets. In Section 3.3.1, we evaluate the effect of integrating MS. In Section 3.3.2, we perform a sensitivity analysis on regularization. In Section 3.3.3, we evaluate the capability of DETexT in different specific chromosomes and add experiments to compare the proposed method with several classical machine learning algorithms.

### 3.1 Experiments on test and training sets of different sizes

To validate the performance of DETexT against an explicit evaluation benchmark, we tested it on a simulated dataset. The simulated data were obtained from the Genome in a Bottle (GIAB) published authentic structural variant marker dataset, a NIST-hosted consortium dedicated to the authoritative characterization of benchmark human genomes, which currently has characterized a pilot genome from the HapMap project (NA12878/HG001) and two son/father/mother trios of Ashkenazi Jewish and Han Chinese ancestry from the Personal Genome Project (Zook et al., 2020). Highly reliable variant annotation files are available from official public data sources and are widely used for the evaluation or testing of variant detections (Poplin et al., 2018; Cameron et al., 2019). We downloaded the candidate variant results for HG002 and its families HG003 (father) and HG004 (mother) as a source for the simulation training dataset. The sample NA12878 (HG001) of the descendants of the CEU trio was downloaded for inference. To construct the training sample dataset, we randomly selected 10,000 sample data from each category (both true variants and false positives) of each original dataset and obtained labels for true positives and false positives by comparing with the original VCFs to form a subset of 20,000 sample data with labels.

**FIGURE 5:**
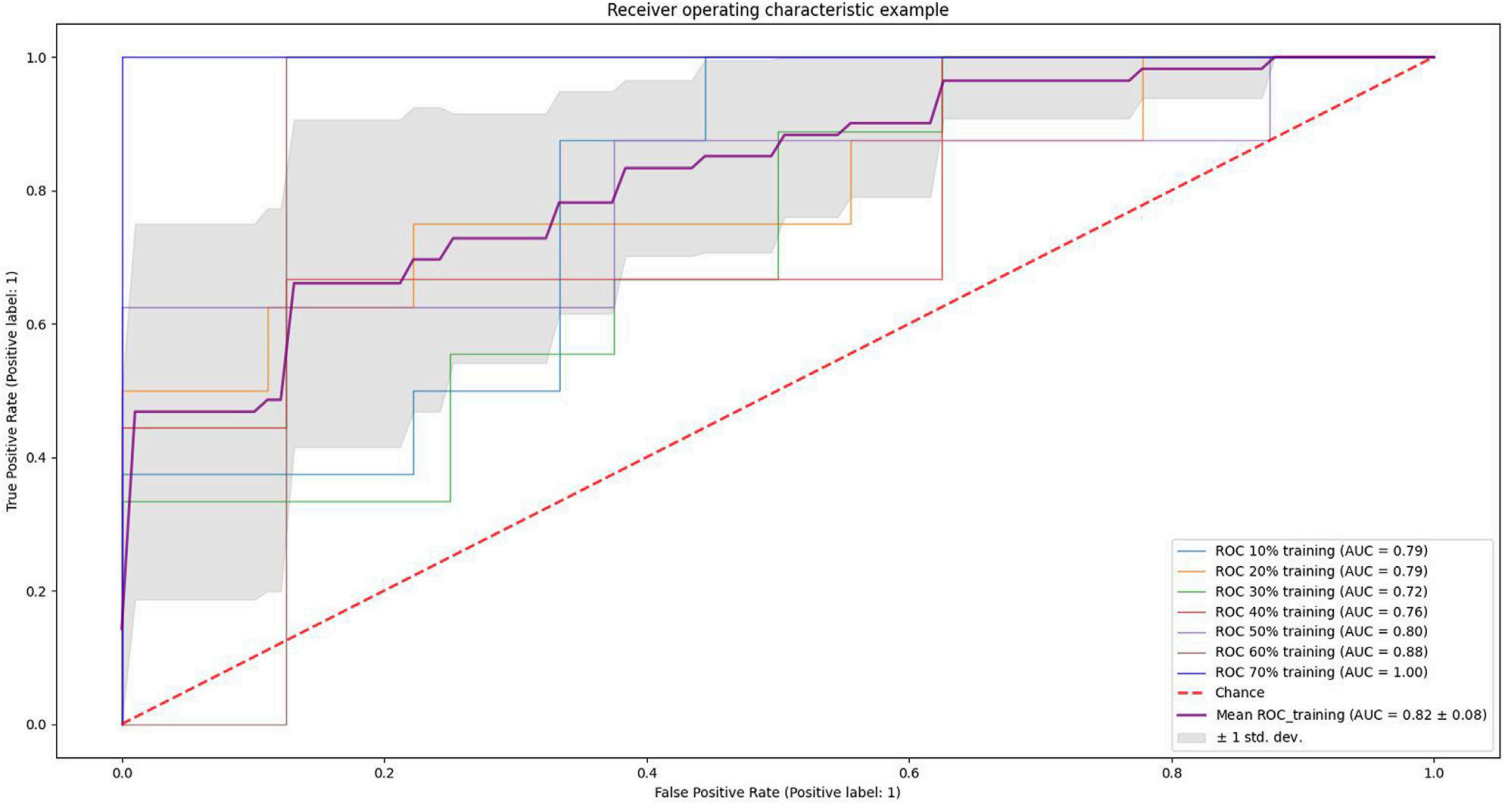
Example of receiver operating characteristics. The light blue curve in the figure indicates the results of the experiment when 10% of the dataset samples are used as the training set and 90% as the test set, light yellow indicates 20% of the dataset is used for training and 80% for testing, light green indicates 30% of the dataset is used for training and 70% for testing, light red indicates 40% for training and 60% for testing, light purple indicates 50% as the training set and 50% as the testing set, brown indicates 60% as the training set and 40% as the testing set, and dark blue indicates 70% of the data as the training set and 30% as the testing set. The curve highlighted in dark purple indicates the average value, and the gray area indicates the value space of the seven results.

We conducted experiments on different proportions of the training and test set. We used scikit-learn’s StratifiedKFold function to slice the training and test set (*n*-fold cross-validation), and the results are shown in Figure 5. The light blue curve in the figure indicates the experimental results when 10% of the samples of the dataset are used as the training set and 90% as the test set, light yellow indicates that 20% of the dataset is used for training and 80% for testing, and light green indicates 30% of the dataset is used for training and 70% for testing. Light red indicates 40% for training and 60% for testing, light purple indicates 50% as the training set and 50% as the testing set, brown indicates 60% as the training set and 40% as the testing set, and dark blue indicates 70% of the data as the training set and 30% as the testing set. The curve highlighted in dark purple indicates the average value, and the gray area indicates the value space of the seven results. It can be seen that the AUC gradually increases as the proportion of the training set increases.

### 3.2 Comparative experiments on simulated data

To simulate data with low read depths, we extract read pairs from the same read pairs in the BAM file to simulate different coverage rates. Based on the position information, one read pair matching the same position is extracted to simulate a ×1 read depth, two to simulate ×2 read depth, and so forth. Once the new BAM/SAM file was obtained, the variant identification was then re-run, and those with the same mark as the real variant are true positives and those with different marks are false positives. We simulated sequencing samples with ×1 read depth and extracted 20,000 candidate mutations each time to form the test data, and a total of eleven simulations were performed to obtain 1 training dataset and 10 test datasets. The reference genome here is the human genome 19. We use classical data sampling methods to balance positive and negative categories: under-sampling a large amount of data in a category (classical easensemble) and over-sampling a small amount of data in a category (classical SMOTE). For each simulated dataset, the positive and negative categories are balanced. We chose accuracy, recall, precision, and F1-score as evaluation criteria. The experimental results for the 10 datasets are shown in Table 2.

**TABLE 2:** Detection performance on simulated datasets.

Datasets	Accuracy	Recall	F1-score	Precision
1	0.8000	0.7700	0.7942	0.8200
2	0.8950	0.8900	0.8945	0.8990
3	0.8350	0.9300	0.8493	0.7815
4	0.8700	0.9000	0.8738	0.8491
5	0.8700	0.9000	0.8738	0.8491
6	0.8300	0.7900	0.8229	0.8587
7	0.7950	0.7700	0.7897	0.8105
8	0.8300	0.7900	0.8229	0.8587
9	0.8483	0.8100	0.8423	0.8773
10	0.8717	0.8367	0.8670	0.8996

We compared DETexT with the well-established and popular SNV detection algorithms MuTect (Cibulskis et al., 2013)*, freebayes* (Garrison and Marth, 2012), and SiNVICT (Kockan et al., 2017). MuTect is a reliable and accurate identification of somatic point mutations in NGS data and is known for sensitive detection in impure and heterogeneous cancer samples. *Freebayes* is a Bayesian genetic variant detector designed to look for small polymorphisms, especially SNPs, indels, MNPs (multi-nucleotide polymorphisms), and complex events (composite insertion and substitution events) with lengths smaller than short-read sequencing alignments. *SiNVICT* is an ultrasensitive detection of single nucleotide variants and indels in circulating tumor DNA, with advanced and promising applications. The comparison results on 10 test datasets are shown in Table 3. The results show that similar methods perform poorly at very low read depths, whereas DETexT achieved over 80% accuracy.

**TABLE 3:** Comparison result with MuTect, freebayes, and SiNVICT.

Accuracy					
Dataset	DeTexT	TextCNN without MS	MuTect	Freebayes	SiNVICT
1	**0.80**	0.51	0.34	0.43	0.29
2	**0.895**	0.52	0.32	0.44	0.33
3	**0.835**	0.45	0.40	0.43	0.42
4	**0.87**	0.56	0.33	0.42	0.44
5	**0.87**	0.52	0.31	0.43	0.42
6	**0.83**	0.54	0.33	0.34	0.32
7	**0.795**	0.49	0.29	0.32	0.29
8	**0.83**	0.45	0.33	0.34	0.36
9	**0.848**	0.51	0.29	0.33	0.25
10	**0.872**	0.49	0.31	0.41	0.33

The best results are highlighted in boldface.

### 3.3 Experiments on the esophageal cancer datasets

We applied DETexT to SNV detection of esophageal cancer (ESCC), the seventh most common and sixth deadliest cancer in the world. The largest ESCC project sequenced 508 ESCC samples by whole-genome sequencing, with an average sequencing depth of ×98 for tumors and ×44 for normal tissues. The 7,630,294 SNVs and indels associated with ESCC cancer species were accurately identified (Cui et al., 2020). We selected the final results published by the project, implanting all 7,454,579 SNVs into the corresponding positions in the reference genome (hg19). We used the NGS simulator GSDcreator (Wang et al., 2019) to simulate the sequence data, which included simulated sequencing errors, amplification bias, unique molecule indices, adapter artifact, quality score distribution, GC content, population polymorphisms, sequencing depth distribution, and insert-size distribution. We simulated paired-end sequencing with 100-bps sequencing read length. We set the sequencing error rate to 0.001, the amplification error rate to 3.0, the template length to (0, 600), and normal bases varying in (33, 36). The sequencing error base was in the interval (7, 19).

**FIGURE 6:**
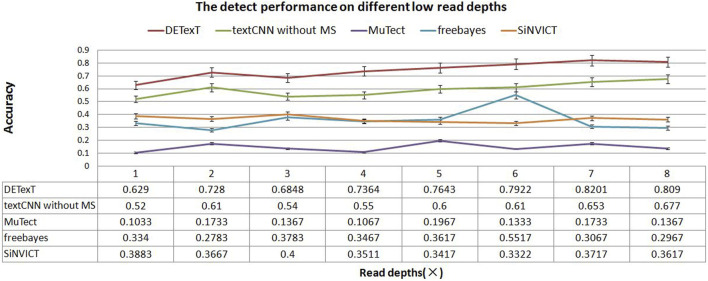
Detection performance at different low read depths. The experimental results of DETexT were compared with those of textCNN without MS, MuTect, freebayes, and SiNVICT on datasets with read depths of 1–8×.

We generated sequencing sample data by seeding the ESCC SNPs into the hg19 reference genome using read pair generation software to simulate sequencing depths of 1–8×. We used DETexT for SNP variant detection discrimination and the candidate variants obtained by the string position matching algorithm constitute the test set in this section. Corresponding to a sequencing read pair depth of 1–8×, 20,000 candidates were selected for each sample to form a total of 8 test sets, and another dataset with 20,000 data was extracted from 5× samples as the training set. In addition, we added a comparison with a textCNN model that does not integrate mutation signatures. The results show that DETexT can accurately detect the SNV as shown in Figure 6, with a 10 percentage points improvement in accuracy compared to the other four methods.

#### 3.3.1 Experiments of mutational signatures

As previously described, the proposed method selects the maximum probability of occurrence in COSMIC MS multiplied by a fixed coefficient and adds it to the previous layer before Softmax to assist in discriminative classification. A total of two sets of experiments were performed to test the effect of mutation signatures. The first experiment was a sensitivity experiment on the coefficients, and the second compared the proposed method with the integration of only the MS probabilities of individual cancers. Here, the training set is the 5× training set presented in Section 3.3, and the test set is a concatenated set of the 1–8× ESCC sample datasets in Section 3.3 (keeping all candidates appearing in the 8 datasets and removing duplicate terms).

The first experiment was a sensitivity experiment of the coefficients. There is a difference in numerical dimensional between the features extracted by the deep network and MS probability, so we added parameters and used the results of multiplying the parameters with the MS probabilities to incorporate the feature results. We conducted parameter comparison experiments and recorded the results of parameter variation in the interval (0,10), as shown in Table 4A. Experiment 1 focused on the incorporation of the multiplied parameters, and we found that the better performance was achieved when the coefficient was 7. Iters denotes the number of iteration in which the model converged.

**TABLE 4:** Experiments of MS and normalization.

(A) Experiment of coefficient selection											
Coefficient	0	1	2	3	4	5	6	7	8	9	10
Iters	2050	1,450	1,400	1,100	2,500	2,500	2,450	1850	300	2,500	1850
AUC	0.9972	0.9988	0.9979	0.9986	0.9950	0.9972	0.9992	**0.9994**	0.9958	0.9966	0.9916
ACC	0.8755	0.8740	0.8522	0.8688	0.8700	0.8744	0.8820	**0.8897**	0.8845	0.8650	0.8840

(B) Experiment of ESCC’s MS integration											
Iters	2000	2000	2000	2000	2000	2000	2000	2000	2000	2000	2000
AUC	0.9255	0.9533	0.9750	0.9577	0.9655	0.9750	0.9776	0.9588	0.9800	0.9827	0.9730
ACC	0.8400	0.8680	0.8400	0.8551	0.8727	0.8791	0.8550	0.8566	0.8544	0.8550	0.8554

(C) Experiment of dropout rate											
Dropout rate	none	0.0	0.1	0.2	0.3	0.4	0.5	0.6	0.7	0.8	0.9
AUC	0.9862	0.9884	0.9906	0.9928	0.9950	0.9972	**0.9994**	0.9983	0.9972	0.9961	0.9862
ACC	0.7979	0.8132	0.8285	0.8438	0.8591	0.8744	**0.8897**	0.8797	0.8697	0.8597	0.7979

(D) Experiment of *l*2 norm											
S	1	2	3	4	5	6	7	10	15	20	30
AUC	0.9972	0.9933	**0.9994**	0.9977	0.9935	0.9910	0.9884	0.9859	0.9834	0.9808	0.9972
ACC	0.8703	0.8800	**0.8897**	0.8851	0.8805	0.8822	0.8797	0.8782	0.8767	0.8753	0.8703

The best results are highlighted in boldface.

**FIGURE 7:**
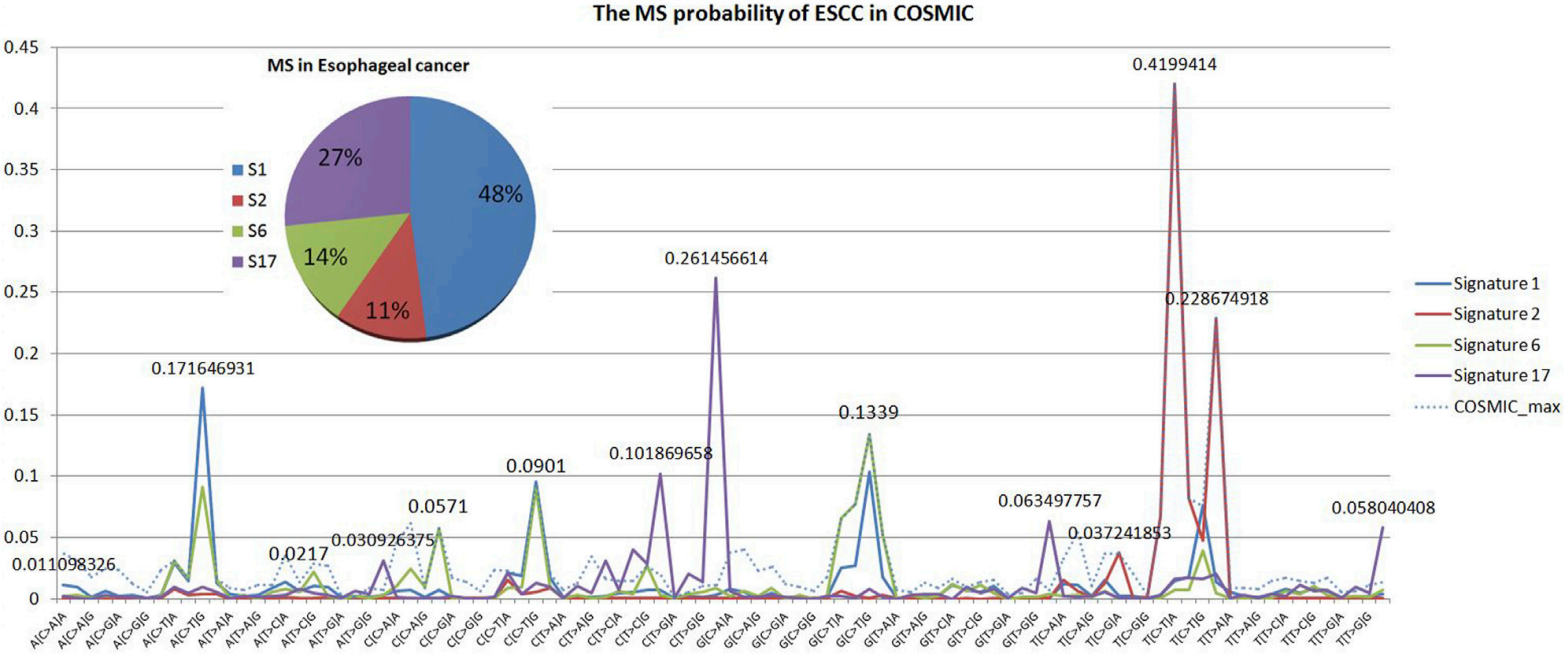
MS probability of ESCC in COSMIC. MS statistics specific to ESCC and comparison with the COSMIC schematic. ESCC has four MSs, COSMIC MS 1, 2, 6, and 17, accounting for the probability statistics of the 96 tri-nucleotide structure results of MS 1, 2, 6, and 17, as shown in the pie. Cosmic_max indicates the maximum value of the cosmic library records corresponding to the 96 tri-nucleotide results.

In addition, we tested the effect of different probability selection methods on the validity of the model in Experiment 2. The results of selecting only MSs associated with ESCC for integration were compared with those of extracting the maximum probability without differentiating cancer types. Our analysis of the literature and data yielded MS specifically for ESCC cancer types as COSMIC MS 1, 2, 6, and 17, with percentages of 48%, 11%, 14%, and 27%, respectively, as shown in the pie chart in Figure 6 (Alexandrov et al., 2013). The ESCC project also showed that 11 MSs (S1–S11) were identified in the 508 WGS cohort, and nine signatures corresponded to mutation signatures in the COSMIC database. S1 and S2 have related to APOBEC (apolipoprotein B mRNA editing enzyme, catalytic polypeptide-like) activity, S3 with DNA mismatch repair deficiency (dMMR), S4 with age, S8 with aristolochic acid, S9 with alcoholic consumption, and S11 with homologous recombination deficiency. S6 was similar to COSMIC signature S17, and recent studies implicated its association with gastric acid reflux (Cui et al., 2020). We plotted the probability results for MS1, 2, 6, and 17 against the maximum probability values in cosmic in Figure 7 and compared the results using the maximum probabilities from the entire cosmic library using the maximum values in MS1, 2, 6, and 17, which are more relevant to ESCC, as shown in Table 4B. The results show that the differences between the two methods are not significant for the reasons that can be seen in Figure 7, where the maximum probability values in MS1, 2, 6, and 17 do not differ significantly from the COSMIC library, but there is also some difference in accuracy from the results.

#### 3.3.2 Experiments of regularization

In total, two common regularization strategies for CNNs are dropout and *l*2 norm constraints. As mentioned earlier, we set the dropout rate (*p*) to 0.5 and the *l*2 norm constraint (s) to 3 based on the sensitivity analysis. Here, we describe the experimental details of the regularization. The “dropout” was applied to the penultimate layer of inputs. We experimented with dropout rate ranging from 0.0 to 0.9, fixing the *l*2 norm constraint to 3. The results are shown in Table 4C. We also report the accuracy achieved when we remove both dropout and the *l*2 norm constraint (i.e., when no regularization is performed), denoted by “None”. In addition, we considered the effect of imposing the *l*2 norm on the weight vectors that parameterize the Softmax function. We recall that the *l*2 norm of a weight vector is linearly scaled to a constraint s when it exceeds this threshold, so a smaller s implies stronger regularization. (Like dropout, this strategy is applied only to the penultimate layer.) We show the relative effect of varying s in Table 4D, where we have fixed the dropout rate to 0.5. From the results, one can see that non-zero dropout rates can help (though very little) at some points from 0.1 to 0.5. But imposing an *l*2 norm constraint generally does not improve performance much. We see that dropout on the convolution layer helps little, and large dropout rate may hurts performance.

**FIGURE 8:**
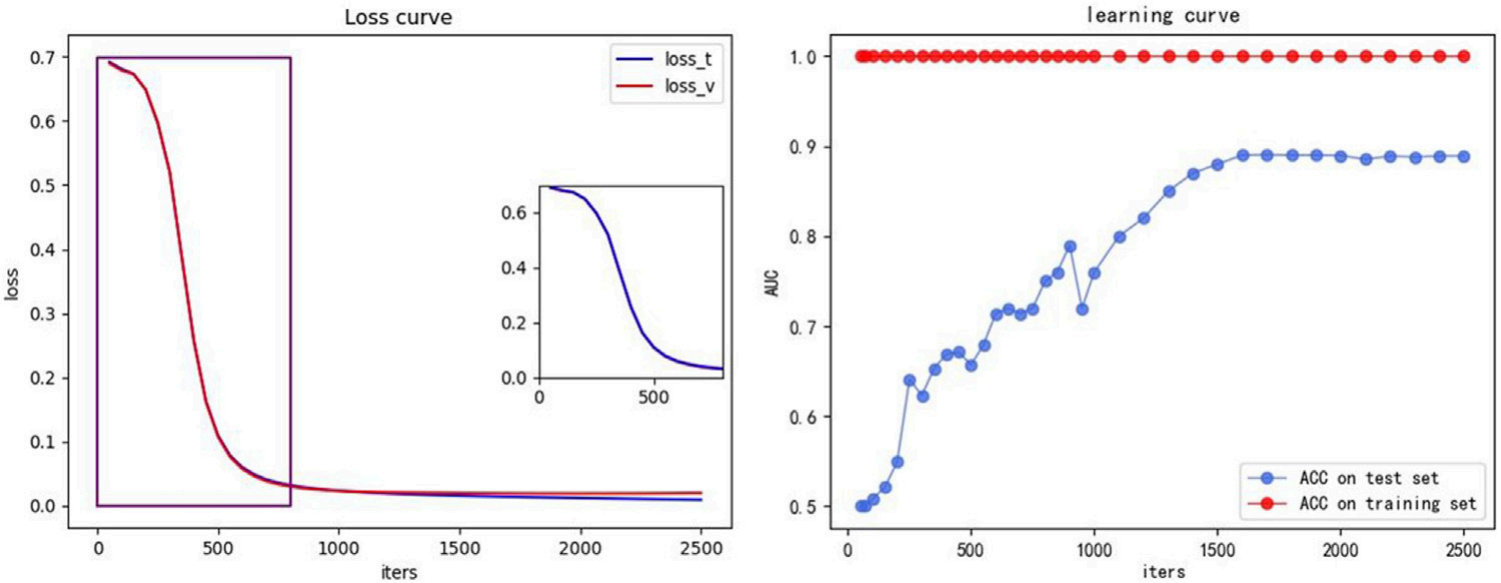
Loss and learning curve. The small graph on the left shows the loss curve, the blue curve (loss_t) indicates the loss of the training set, and the red curve (loss_v) indicates the loss of the test set. The small graph on the right shows the learning curve, the red curve (ACC on training set) indicating the ACC of the training set, and the blue curve (ACC on test set) indicates the ACC of the test set.

We also plotted the loss curves and the learning curves, as shown in Figure 8, to demonstrate that our model can converge quickly without overfitting. The training set is same as the Section 3.3.1, being the ×5 training set introduced in Section 3.3 and the test set is the concatenated set of 1–8× ESCC sample datasets from Section 3.3 (retaining all candidates appearing in the eight datasets and removing duplicate terms). The iters is the number of iterations, indicating the number of iterations for which the model converges. The small graph on the left shows the loss curves, with the blue curve (loss_t) indicating the loss of the training set and the red curve (loss_v) indicating the loss of the test set. The small graph on the right shows the learning curve, with the red curve (ACC on training set) indicating the ACC of the training set and the blue curve (ACC on test set) indicating the ACC of the test set.

#### 3.3.3 Experiments on different chromosomes

**FIGURE 9:**
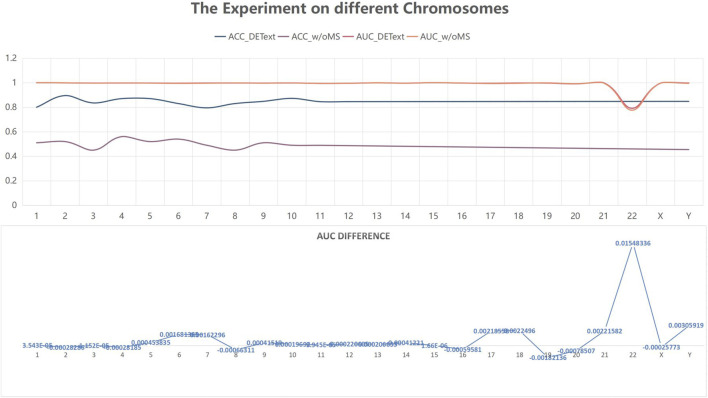
Experiment on different chromosomes. The effect of mutational signatures on each chromosome. The overall performance of the method was good, whereas the effects of mutational signatures on different chromosomes are slightly different.

To further explore the effectiveness of the proposed method, we compared its performance on different chromosomes, as shown in Figure 9 and Table 5. We randomly selected 20,000 reads from each chromosome data on the 1–8× dataset described in 3.3 to form 24 test datasets, additionally extracted 20,000 data on chromosome 2 as the training data set, and calculated the performance of the model on these 24 test sets. In addition, we tested the proposed method with another pooling algorithm and other machine learning algorithms, including decision trees, gradient boosted decision trees (GBDT), random forests, and support vector machines (SVM). The accuracy results are shown in Table 6. The results show that DETexT has good overall performance, with slightly different results for mutation signatures on different chromosomes. On chromosomes 8, 16, and 19, the effect of MS tends to be negative, whereas on the other chromosomes, positive effects of integrated MS can be seen, especially on chromosome 22.

**TABLE 5:** Detection performance on a real dataset and shown in each chromosome.

Chromosome	Accuracy	Recall	F1-score	Precision
1	0.8500	0.8499	0.8499	0.8500
2	0.8470	0.8441	0.8456	0.8470
3	0.8459	0.8420	0.8439	0.8459
4	0.8475	0.8451	0.8463	0.8475
5	0.8470	0.8441	0.8455	0.8470
6	0.8462	0.8425	0.8443	0.8462
7	0.8467	0.8434	0.8451	0.8467
8	0.8474	0.8450	0.8462	0.8474
9	0.8465	0.8430	0.8447	0.8465
10	0.8478	0.8455	0.8466	0.8478
11	0.8453	0.8406	0.8429	0.8453
12	0.8465	0.8431	0.8448	0.8465
13	0.8480	0.8459	0.8469	0.8480
14	0.8463	0.8426	0.8444	0.8463
15	0.8500	0.8500	0.8500	0.8500
16	0.8463	0.8427	0.8445	0.8463
17	0.8443	0.8388	0.8415	0.8443
18	0.8483	0.8465	0.8474	0.8483
19	0.8476	0.8451	0.8463	0.8476
20	0.8436	0.8373	0.8404	0.8436
21	0.8472	0.8443	0.8457	0.8472
22	0.7254	0.8299	0.7654	0.7101
*X*	0.8470	0.8442	0.8456	0.8470
*Y*	0.8448	0.8397	0.8423	0.8448

**TABLE 6:** Comparison experiment with average_pooling and traditional machine learning algorithms.

Chromosome	Baseline	DETexT 1Max_pooling	Average_pooling	Random forest	SVM	GBDT
1	0.7098	0.8500	0.8029	0.6270	0.5347	0.5962
2	0.7312	0.8470	0.7856	0.6320	0.5150	0.5170
3	0.7526	0.8459	0.7683	0.6370	0.4953	0.4378
4	0.7214	0.8475	0.7510	0.6420	0.4756	0.4183
5	0.7051	0.8470	0.7871	0.6470	0.4559	0.5927
6	0.71826	0.8462	0.7881	0.6520	0.4362	0.4184
7	0.71634	0.8467	0.7891	0.6570	0.4165	0.4662
8	0.71442	0.8474	0.7900	0.5620	0.3968	0.6674
9	0.7125	0.8465	0.7910	0.6670	0.3771	0.5962
10	0.71058	0.8478	0.7920	0.6720	0.3574	0.5170
11	0.713687	0.8453	0.7930	0.6770	0.3377	0.4378
12	0.713921	0.8465	0.7940	0.6820	0.3180	0.4183
13	0.714155	0.8480	0.7950	0.5870	0.2983	0.4370
14	0.71439	0.8463	0.7960	0.6920	0.2786	0.4534
15	0.714624	0.8500	0.7970	0.6970	0.2589	0.5326
16	0.714858	0.8463	0.7980	0.7020	0.5150	0.6038
17	0.715092	0.8443	0.7989	0.7070	0.4953	0.6830
18	0.715327	0.8483	0.7999	0.6370	0.4756	0.7046
19	0.715561	0.8476	0.8009	0.6420	0.4559	0.6674
20	0.715795	0.8436	0.8019	0.6470	0.4362	0.5962
21	0.71603	0.8472	0.8029	0.6520	0.4165	0.5170
22	0.716264	0.7254	0.8039	0.6570	0.3968	0.5134
*X*	0.716498	0.8470	0.8049	0.6620	0.3771	0.4183
*Y*	0.716732	0.8448	0.8059	0.6670	0.3574	0.4370

## 4 Discussion and conclusion

This work focuses on SNV detection for low read depths in NGS data, but can also be used for high depth calls, and performs comparatively well compared to other advanced methods with a big emphasis on utilization when looking at low depth WGS. Compared with existing methods based mainly on extracting statistical results from correlated features of read pairs, 1) the proposed differential representation ensures maximum utilization of base sequence information and differential information on the one hand and minimizes storage and operation costs on the other. 2) The integration of the mutational signature is enlightening for distinguishing true SNV from false positives. We have conducted extensive experiments, and the results show that the proposed method can accomplish SNV detection at low read depth with low cost and high efficiency.

Furthermore, although the structure and parameters of the proposed method are relatively clear, the deep learning model is still a black box and/or the interpretability of the model is worth investigating. Our study highlights the importance of using mutational signatures, and in experiments testing the effect of MS on different chromosomes, we have noticed that the effects of MS on different chromosomes was different. We cannot assert that the effect of the proposed method will not change at all in other cancer types or new datasets, which suggests that more work on the model with larger datasets is still worth exploring. We aim to collect more data to explore the effect of MS on variant detection and expand the cancer species data in the future work.

## Data Availability

The original contributions presented in the study are included in the article/Supplementary Material; further inquiries can be directed to the corresponding author.

## Abbreviations

ACC, accuracy; COSMIC, Catalogue Of Somatic Mutations In Cancer; CNN, convolutional neural network; cfDNA, cell-free circulating DNA; ctDNA, cell-free tumor DNA; MS, mutational signature; NMF, nonnegative matrix factorization; NGS, next-generation sequencing; PCAWG, Pan-Cancer Analysis Of Whole Genomes; ROC, receiver operating characteristic; ReLU, rectified linear unit; SNV, single nucleotide variant; SAM, sequence alignment map; SV, structural variant; TCGA, the cancer genome atlas; VAF, variant allelic frequency; VCF, variant calling format.
