# Supplementary figures and images for: DETexT: An SNV detection enhancement for low read depth by integrating mutational signatures into TextCNN
Source: Front Genet. 2022 Sep 28;13:943972. doi: 10.3389/fgene.2022.943972 (PMC9554618; doi:10.3389/fgene.2022.943972)

## Supplement

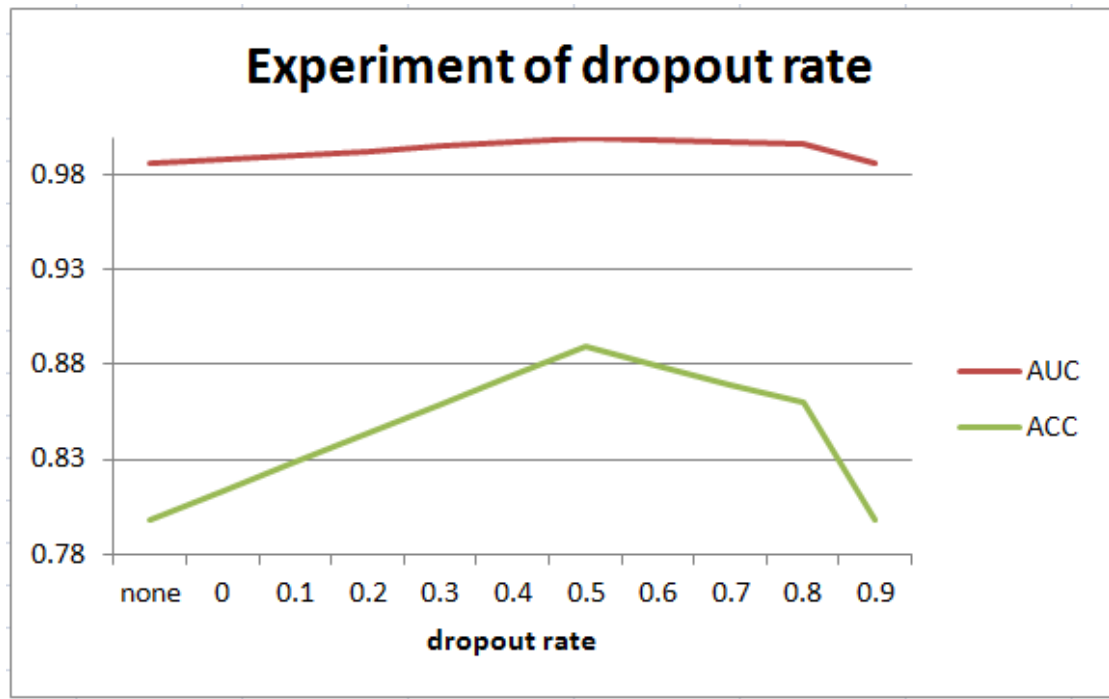

## Experiment of $l_2$ norm

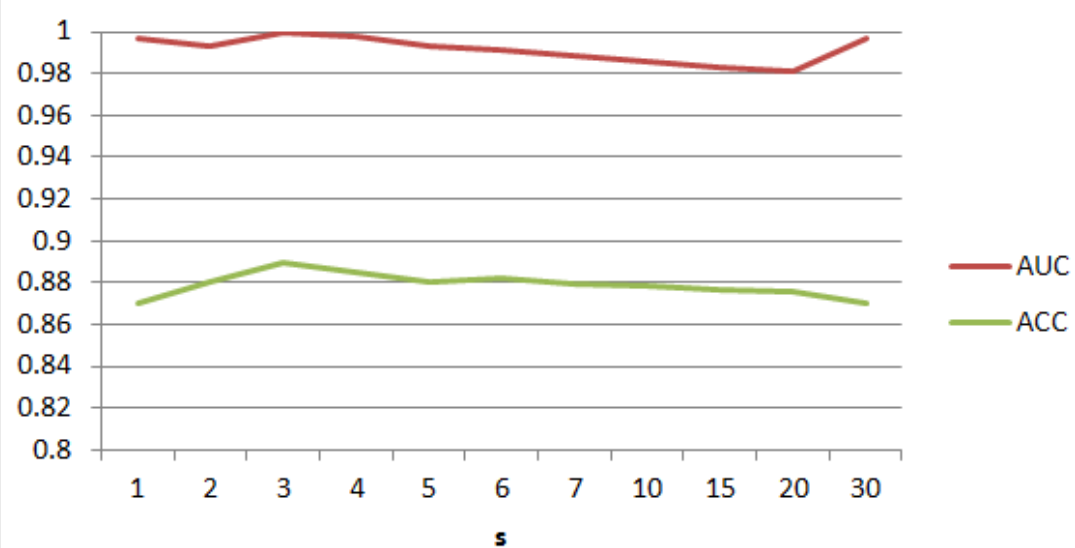

Supplement: Supplementary file 1 [file DataSheet1.PDF]
